# HIV self-testing in cis women in Canada: The GetaKit study

**DOI:** 10.1177/17455057251322810

**Published:** 2025-03-31

**Authors:** Lauren Orser, Alexandra Musten, Hannah Newman, Molly Bannerman, Marlene Haines, Jennifer Lindsay, Patrick O’Byrne

**Affiliations:** 1 School of Nursing, University of Ottawa, Ottawa, ON, Canada; 2 Women and HIV/AIDS Initiative, Toronto, ON, Canada

**Keywords:** HIV self-testing, women, HIV, healthcare access

## Abstract

**Background:**

In light of ongoing HIV diagnoses among cis women, despite decreases in other populations, such as men who have sex with men, various testing approaches, including HIV self-tests are being targeted at cis women as a means of identifying undiagnosed HIV infections and of linking those with positive test results to care. Little, however, is known about risk characteristics of cis women who access HIV self-tests in Canada.

**Objectives:**

Our objectives were to examine demographic characteristics, risk factors, and test results of cis women who obtained HIV self-tests through the HIV self-testing platform, GetaKit.ca.

**Design:**

GetaKit.ca was an observational cohort study that provided free HIV self-tests to Canadians with reported risk factors for HIV acquisition.

**Methods:**

We completed an analysis of cis women who ordered HIV self-tests from GetaKit.ca between April 1, 2021 and May 31, 2023. Data analysis involved tabulating frequencies and means, plus chi-square calculations to determine significant differences between cis women and cis men who obtained HIV self-tests.

**Results:**

During the study period, 7420 orders for HIV self-tests were made through GetaKit.ca; 22% of these orders were made by cis women. Compared to cis men, cis women had significantly higher reported rates of injection drug use and significantly lower reported rates of prior sexually transmitted infection testing, HIV testing (with more cis women indicating their last HIV test was more than 12 months ago), and reporting HIV self-test results. Despite this, we found no differences in the number of cis women with a positive HIV self-test compared to cis men (positivity rate of 0.2% versus 0.3%, respectively).

**Conclusion:**

Our findings showed less overall uptake of HIV testing in cis women, despite matched risks and positive test results. Future interventions to engage cis women in HIV testing should include increased access points for HIV self-tests and enhanced linkage to care pathways to HIV pre-exposure prophylaxis or HIV treatment.

## Introduction

In Canada,^1^ and more locally, Ontario,^2^ cis gender women (henceforth referred to as women or females) are considered an HIV priority group due to population-specific epidemiology and social and structural inequities (including individual and partner specific risk behaviors), which can increase the risk of HIV acquisition for these persons.^3,4^ This is an important point because, although HIV has to date disproportionately affected cis gender men (henceforth referred to as men or males), specifically men who have sex with men (MSM),^3^ in recent years, the number of new infections among MSM has decreased,^5^ but no real change in the number of new HIV diagnoses has occurred among females. The outcome of this change in overall diagnosis numbers is that females have begun to account for a larger proportion—but not necessarily a larger total number—of new HIV diagnoses each year. Epidemiologic data suggest that females accounted for 33% of new HIV diagnoses in Canada in 2022, which is an increase from 28% in 2021.^1,3^ In Ontario (the most populous province in Canada and where this study originated), females have consistently accounted for 20%–25% of all new HIV diagnoses over the past 10 years.^2^

Because testing is the entry point to the HIV prevention and care cascade^3^ (in that, a positive test result warrants treatment and a negative test result is an opportunity for prevention, including pre-exposure prophylaxis (PrEP)), HIV self-testing has been lauded as a new way to engage persons with undiagnosed HIV.^6^ The importance of HIV self-testing has been further emphasized as an access point for individuals who encounter challenges when seeking testing, including women.^7,8^ Such barriers may relate to potential stigma from healthcare providers, requirements to disclose certain risk practices to providers in order to obtain testing, and difficulties in accessing clinic locations (e.g., time required to attend clinical visits, transportation to and from clinics).^9,10^ Despite women being a potential key population for HIV self-testing, very little is known about women who use this testing modality in Canada. Indeed, we were unable to identify any literature that focused exclusively on women in Canada. All studies reported on smaller groups of women, without doing in-depth analyses of this population. As such, due to the potential utility of HIV self-testing for women, yet an absence of literature, we reviewed and now present on data about women from the GetaKit.ca study, which was a website-based research project through which persons in Canada were able to register, complete an online HIV risk assessment,^11^ and, when indicated according to clinical guidelines,^12–14^ obtain free HIV self-tests based on their reported risk factors. Previous research involving GetaKit participants has been published,^15–19^ focusing on rates of invalid self-test results, acceptability of HIV self-tests, linkage to care, and uptake and outcomes among sub-groups of participants who identified as MSM, persons from African, Caribbean, or Black communities, and first-time testers. We focused on women here to get more in-depth data on this population to better inform (1) our understanding of HIV self-testing within this group of persons and (2) future health interventions to increase HIV testing and linkage to preventative and treatment health services for women.

## Methods

### The GetaKit study

GetaKit.ca was a prospective observational study^20^ that provided free HIV self-tests to persons with reported risk factors for HIV acquisition. The study was operated by a team of nursing researchers at the University of Ottawa, School of Nursing. GetaKit.ca launched in Ottawa, Ontario (a city of ~1 million residents) in July 2020 and expanded across Ontario (population of ~18 million) starting on April 1, 2021 with services available in English, French, Spanish, and simplified Chinese. Full Ontario-wide distribution began in July 2021 and pan-Canadian distribution started in November 2022. On June 1, 2023, GetaKit.ca began offering full sexually transmitted infection (STI) testing for syphilis, hepatitis C, gonorrhea, and chlamydia.

### Recruitment

Recruitment occurred via online promotion (Internet, social media) and through in-person community events. Promotion also occurred within local AIDS service organizations across Ontario, including through posters at their venues and during outreach community talks. To be eligible, potential participants had to be at least 16 years of age and have risk factors for HIV acquisition (through sexual or drug use practices); exclusion criteria included already being diagnosed with HIV, a diagnosed bleeding disorder, and enrollment in an HIV vaccine trial.

To obtain an HIV self-test, people had to visit the study website (GetaKit.ca), review and digitally sign the online consent forms, register using a name and contact information, complete an online HIV risk assessment developed by the GetaKit team^11^ (based on the United States CDC guideline),^14^ consent to obtaining the self-test, and place their order (see Supplemental Appendix 1). Vertical flow qualitative fingerstick blood-based HIV self-tests were then made available to eligible participants through mail-out delivery to a participant’s fixed address or via curbside pickup at local AIDS service organizations, pharmacies, and sexual health clinics. We used the BioLytical INSTI^®^ HIV self-test, which was the only self-test approved for use in Canada at the time of this study.

The GetaKit.ca risk algorithm stratified participants based on their reported risk factors^12–14^ such that (1) they were not offered any HIV self-tests if they did not have any risk factors (i.e., they did not report recent or prior sexual contact or drug use) or if they were already diagnosed with HIV, (2) they received an offer of one HIV self-test if they did not have exclusion criteria and reported any risk for HIV acquisition, and (3) they were offered two HIV self-tests if they reported being a sexual or drug use contact of someone with transmissible HIV. Participants received two automated email reminders to submit their HIV self-test results, but were not obligated to report their self-test result. Registered nurses working for the GetaKit study reached out directly by phone to anyone who reported a positive self-test result and directly connected them to in-person confirmatory testing and support services.

### Data collection

Data collection occurred through the GetaKit.ca website and were compiled per order. These data included all information collected from participants’ risk assessments, such as demographics (sex/gender, race/ethnicity, sexual orientation), risk practices (recent or prior sex/drug use behavior), past medical histories (prior STI/HIV testing and diagnoses), and reported HIV self-test results (as negative, positive, invalid, or prefer not to report).

Data from the risk assessment completed at each order were housed in the GetaKit.ca database. We extracted the full dataset for the period of April 1, 2021 to May 31, 2023 and filtered results to include eligible orders made by participants who identified as cis women. This period excluded the pilot phase and the expansion phase to offer STI testing. These 26 months thus cover the time when only HIV self-tests were available through the GetaKit.ca study.

### Statistical analysis

Data analysis involved descriptive statistics (counts, means, frequencies) and chi-square calculations to identify significant differences among our participants who identified as cis women. Calculations were completed in Excel. We selected the comparator for these chi-square analyses to be cis men, as they were the largest group in our overall cohort and the group for which most information is known regarding HIV self-testing. We selected a *p*-value of 0.05 *a priori* to determine significance.

### Ethics

The study was funded by the Ontario HIV Treatment Network, Public Health Ontario, and the Public Health Agency of Canada. These funders had no influence on data collection or data analysis. The research ethics board at the University of Ottawa approved this project. We followed the STROBE guidelines^21^ when preparing the manuscript (see Supplemental Appendix 2).

## Results

### Participant demographics

From April 1, 2021 to May 31, 2023, there were 7420 orders for HIV self-tests placed through GetaKit.ca, of which 79% (*n* = 5852/7420) were eligible. Of eligible orders, 22% (*n* = 1277/5852) came from cis women and 67% (*n* = 3934/5852) from cis men (as noted above, for brevity, we refer to these two groups as women or females and men or males, respectively). Another 2.5% (*n* = 145/5852) of orders were from persons who identified as trans and 7% (*n* = 379/5852) from persons who identified as nonbinary. Among orders from participants who identified as women (*n* = 1277), 71% (*n* = 909/1277) were heterosexual and 24% (*n* = 301/1266) were gay, bi, lesbian, or women who have sex with women. Additionally, 41% (*n* = 490/1187) of these women reported being White and 29% (*n* = 340/1187) indicated that they were of African, Caribbean, or Black ethnicities. Female participants in this study were a mean age of 31 years old and 59% (*n* = 711/1202) were born in Canada. Overall, approximately 98% of orders were placed using the English-language interface and 96% (*n* = 5617/5852) of orders came from Ontario; for female participants, 94% (*n* = 1202/1277) reported that they lived in Ontario. See Table 1 for an overview of the sample.

**Table 1.** Characteristics of women who accessed HIV self-tests through GetaKit.ca.

Category	Total number	*n*	%
Province of residence	1277		
Ontario		1202	94.1
Outside of Ontario		75	5.9
Sexual orientation	1277		
Heterosexual		909	71.2
LGBTQIA2S+		301	23.6
Race/ethnicity	1187		
White		490	41.3
African, Caribbean, or Black		340	28.6
Southeast / East Asian		170	14.3
Arab / West Asian		65	5.5
Latinx		51	4.3
Indigenous		34	2.9
South Asian		37	3.1
Sex work	1177		
No		1082	91.9
Yes		95	8.1
Injection drug use	1182		
No		1078	91.2
Yes		104	8.8
Prior HIV/STI testing	1177		
No (first-time testers)		465	39.5
Yes		721	60.5
Prior HIV/STI diagnoses	712		
No		513	72.1
Yes		199	27.9
Which prior STI diagnoses	193		
Chlamydia		156	80.8
Gonorrhea		32	16.6
Syphilis		4	2.1
HIV		1	0.5

STI: sexually transmitted infection.

### Risk behaviors

For risk factors, 9% (*n* = 104) of female GetaKit.ca participants reported injection drug use (recent or prior) and 8.1% (*n* = 95) reported participating in sex work (recent or prior). This proportion of injection drug use was higher among women (9%) compared to men (7%) (*p* = 0.03, χ^2^ = 4.97); the reported rate of sex work meanwhile was not significantly different between women and men at 8% and 7%, respectively (*p* = 0.33, χ^2^ = 0.91). There were also no differences in the reported rates of being an STI/HIV contact between our female (3%) and male (4%) participants (*p* = 0.12, χ^2^ = 2.38), nor were there differences in the reported rates of having sexual partners with risk factors for HIV acquisition (*p* = 0.34, χ^2^ = 0.91, female: 31% compared to male: 33%). See Table 2 for all chi-square analyses.

**Table 2.** Chi-square analyses comparing characteristics of women and men.

Characteristics	Sex		χ^2^	*p*-Value
	Female	Male		
Injection drug use				
Yes	104	260	4.97	0.02
No	1078	3528		
HIV/STI contact				
Yes	38	160	2.38	NS
No	1145	3635		
Sexual partner with HIV risk factors				
Yes	366	1233	0.91	NS
No	811	2551		
Sex work				
Yes	95	273	0.96	NS
No	1082	3511		
Prior HIV/STI testing				
Yes	712	2404	3.97	0.04
No	469	1382		
Last HIV test				
>12 months ago	294	772	49.78	<0.001
<12 months ago	272	1391		
Last STI test				
>12 months ago	281	905	0.39	NS
<12 months ago	349	1190		
Prior HIV/STI diagnoses				
Yes	199	747	2.57	NS
No	512	1652		
Reported self-test result				
Yes	736	2770	71.91	<0.001
No	541	1162		
Positive HIV self-test (total ordered)				
Yes	3	12	0.17	NS
No	1274	3922		
Positive HIV self-test (total reported)				
Yes	3	12	0.009	NS
No	733	2758		

STI: sexually transmitted infection. NS: not significant.

### Healthcare access

For healthcare access, 61% (*n* = 712/1177) of female GetaKit.ca participants reported prior STI testing. This rate of testing among female participants (61%) was significantly lower than among male participants (64%) (*p* = 0.046, χ^2^ = 3.97). There was, however, no difference in the reported rates of prior STI testing within the last 12 months between women (45%, *n* = 281/630) and men (43%, *n* = 905/2095) (*p* = 0.53, χ^2^ = 0.39). Among women who had previously tested for STIs, 28% (*n* = 199/712) reported a past STI diagnosis, of which the most commonly reported diagnoses were chlamydia (81%), followed by gonorrhea (17%). There were no differences in the reported rates of prior STI diagnoses among women (28%, *n* = 142/520) compared to men (32%, *n* = 747/2399) (*p* = 0.11, χ^2^ = 2.57). Notably, the rates of prior STI diagnoses did not differ between female participants who identified as heterosexual (27%, *n* = 142/530), compared to those who identified as gay, bi, lesbian, or as women who have sex with women (32%, *n* = 57/179) (*p* = 0.19, χ^2^ = 1.69). For HIV testing, only 59% (*n* = 695/1177) of women reported that they had previously done such testing—which means that 1.4% (*n* = 17) of our female participants who had completed STI testing reported no prior HIV testing. Having completed HIV testing more recently was also lower among women compared to men, with 52% (*n* = 294/566) of women who had previously done HIV testing reporting that their last test was more than 12 months ago, compared to 36% (*n* = 772/2163) among men.

### Test outcomes

For test outcomes, 58% (*n* = 736/1277) of women reported their HIV self-test result back to GetaKit.ca, which was significantly lower than the 70% (*n* = 2770/3932) of men who reported a test result (*p* < 0.001, χ^2^ = 71.91). Despite fewer results being reported back to GetaKit.ca for women compared to men, the overall positivity rate was no different between women (0.2%, *n* = 3/1274) and men (0.3%, *n* = 12/3934) (*p* = 0.68, χ^2^ = 0.17). It was even more similar when controlled for reporting rates: the positivity rate was 0.4% among the 736 women who reported their HIV self-test results, and 0.4% among the 2770 men who reported their self-test results (*p* = 0.92, χ^2^ = 0.009). All positive HIV self-tests from this cohort were confirmed by serology to be true positive results and were directly linked to care by GetaKit nurses.

## Discussion

In this article, we reported on HIV self-test distribution to women in Canada through GetaKit.ca from April 1, 2021 to May 31, 2023 and found lower uptake of HIV testing among women compared to men, despite matched HIV positivity rates. Compared to male participants, female GetaKit.ca participants reported more injection drug use, but less prior HIV testing overall and less prior HIV testing within the last 12 months among those who had done testing; they were also less likely to report their HIV self-test results to GetaKit.ca. In contrast, we did not identify differences between our female and male participants regarding risk factors (e.g., being an STI/HIV contact or sex work), or in the rates of prior STI diagnoses or for HIV self-test positivity rates. There were also no differences in the rates of prior STI diagnoses between women who identified as heterosexual and women who identified as gay, bi, lesbian, or as a woman who have sex with women.

Our data thus suggest that women who accessed GetaKit.ca had decreased access to HIV testing, compared to men, despite having the same rates of reported prior STI diagnoses and the same HIV positivity rates. Among women who had previously been tested, more also reported being tested longer ago. This finding is reinforced by provincial laboratory data in Ontario,^22^ which show that males had higher rates of HIV testing in 2021. Notably, this finding about less previous testing did not apply to STI screening, suggesting that this lack of access to testing truly only applies to HIV. It does appear that women are seeking sexual health services, but—for a number of intersectional reasons, including stigma, cultural barriers, geographic location, low perceived risk for HIV, lack of knowledge related to HIV, limited access to healthcare, fears around disclosure, experiences of violence^9,10^—are not completing, or are not able to complete, laboratory-based HIV testing.

Considering the recent changes in HIV epidemiology^2,3^—in that, women have begun to account for a larger proportion of new diagnoses in recent years—ensuring access to testing among women needs to be prioritized. Indeed, making testing available is likely one of the easiest ways to achieve the UNAIDS 95-95-95 targets,^23^ in which 95% of persons living with HIV are diagnosed, 95% of persons with diagnosed HIV are in care, and 95% of persons receiving HIV care achieve viral suppression. Regardless of where or how an HIV exposure might have occurred, testing is important because it is the entry point to HIV treatment, and, without it, people remain undiagnosed and continue to have transmissible infections.^1,23^ A lack of access to testing also means decreased access to prevention services because testing is simultaneously the entry point for interventions like PrEP.^1,23^ When someone tests positive for HIV, they should be linked to treatment and care; when they test negative, they should be informed about retesting and be offered PrEP if risks are ongoing; this includes women.^24,25^ With less testing (and as a corollary, less PrEP use) among women, it is unsurprising that the numbers of new HIV diagnoses have not decreased among women, in contrast to the decreases that have been observed among men (mostly—but not exclusively—related to PrEP use among gay men).^3,5^

We do not, however, interpret our data to mean that access to HIV testing should be unrestricted. HIV self-testing can yield false positive results,^26–28^ and while these would be ruled out with reflex confirmatory testing and repeat screening, false positive results are not without potential harms. While follow-up testing to rule out false positive results is usually prompt, this situation can be highly distressing for persons while they wait for these new test results.^29,30^ At the same time, however, if we are serious about achieving the UNAIDS 95-95-95 targets by 2030, we will need to increase testing among women—but need to do so systematically and in ways that maximize the pre-test probability of diagnosis and minimize the harms of test errors. The problem that our results suggest is that the women who accessed GetaKit.ca did have risk factors for HIV acquisition^12,13^ and should have been tested, but heretofore were not. These women had no differences in their reported rates of prior STI testing (and diagnoses); they did though have lower reported rates of previous HIV testing. Reinforcing the need to test women is that our positivity rates matched between female and male participants, which suggests that targeted screening (i.e., based on reported risk factors) was able to successfully diagnose individuals—who might have otherwise been unaware of their status—and link them to treatment and care. Because most people who complete HIV testing will test negative,^31^ risk-based screening algorithms for self-test distribution may be a useful approach to target HIV testing at women with elevated risk factors for HIV infection and to help reduce the number of undiagnosed infections that continue to occur within subpopulations of women.

Another important finding from this study is that GetaKit.ca (and other online risk-based systems like it) can be a means to appropriately increase HIV testing among women. Using digitized risk assessment algorithms,^11^ GetaKit.ca was able to ensure HIV testing occurred for women who reported no prior testing—despite having sought and obtained STI testing services before. Online systems thus may be one important tool in our collective efforts toward achieving the UNAIDS 95-95-95 targets^23;^ in that, these systems can offer testing in such a way that the positivity rates among women and men match. This finding is important considering that, in Ontario in 2021, the HIV test positivity rates^22^ from the laboratory were 0.126% for males and 0.033% for females (or, nearly four-times higher among males). As such, GetaKit.ca helped target testing to women with the same rate of undiagnosed infection as we observed among men, and this positivity rate of 0.3%–0.4% was three- to four-times higher than what was observed in the provincial laboratory system in Ontario.^22^

Given the lack of data specific to HIV testing among women in Canada, it is difficult to determine why there were such marked differences in positivity rates between women who accessed HIV testing through GetaKit.ca compared to conventional laboratory testing. We can however speculate. One possible reason could relate to differences in risk screening approaches in clinical settings, as from the Ontario testing data, 70% of laboratory-based HIV tests were completed on heterosexual women with no identified risk factors for HIV infection.^22^ It is equally possible that these differences relate to ease of using an online platform to access testing and comfort to disclose risk practices using computer-assisted sexual health interviews compared to when these discussions are posed by a healthcare provider in-person.^32^ Such online systems may also facilitate access to testing; in that, instead of women having to attend clinics, through GetaKit.ca, we delivered self-tests directly to person’s fixed addresses by mail. Perhaps this mail-out approach was a success factor. In any case, more research is required to better understand why women – and more specifically women with undiagnosed HIV – sought out HIV testing via self-testing over laboratory-based approaches.

A final noteworthy finding was that we observed equal rates of prior STI diagnoses in queer and heterosexual women. While research finds that many queer women and healthcare providers often believe that heterosexual women are at higher risk,^33,34^ our data (based on self-report) suggest that this may be a misconception. Further research is required to better understand risk among women who do not identify as heterosexual to further our understandings about the sexual health needs of diverse groups of women. Similar analyses are required for trans women, as well as for nonbinary persons assigned female at birth. If our goal is to truly achieve the UNAIDS 95-95-95 goals,^23^ we must continue to have better understandings about, and testing interventions for, the broader groups of people who do, and do not, identify as women. Without this, we will only exacerbate inequities among trans and non-binary persons.

### Limitations

These results must be interpreted considering certain limitations. First, our data arose from self-report. We did not have objective laboratory data for prior test histories or results (although we did have confirmatory serologic testing results for the reactive HIV self-tests—all of which were confirmed as positive). Self-report can be subject to recall bias and courtesy bias,^35^ when people withhold information that is less socially desirable. While these factors likely influenced our results, they would equally apply to any form of STI/HIV risk assessment, as these always rely on patient self-report. Our data collection thus matches the current standard.^14^ Second, our results may not be transferable, as they arose almost exclusively from Ontario and exclusively from women who accessed a website to obtain an HIV self-test. These women also likely had more resources than those who did not access the site, such as a working phone, laptop, Internet access, and a home/fixed address. Future research needs to evaluate HIV self-testing among women in other jurisdictions and among women who do not have Internet access, digital literacy, or who are underhoused. Further research is also required from jurisdictions where HIV testing is not free. In such areas, the rates of prior HIV testing may be even lower than what we identified in this cohort. Third, we did not have any test results for other STIs—which could have helped better establish the degree of STI/HIV risk among our participants. Future research needs to include full STI testing. Fourth, our analysis was restricted to chi-square analyses of a large dataset, from which statistical significance may have arisen based on numerical differences that are of little clinical and/or public health significance. While this may have influenced our findings, many analyses are subject to the same limitations, and it is through discussion of the data that we determine its relevance. Finally, our risk assessment did not screen for reported experiences of intimate partner violence or survival sex, which (1) are associated with increased risk of HIV acquisition^36^ and (2) may have affected decisions to engage in HIV self-testing over laboratory-based HIV testing.

## Conclusion

In Canada, women now account for a larger proportion of new HIV diagnoses in recent years. Because testing is the entry point to the HIV care cascade (and the entry point for treatment and prevention services), understanding women’s access to this service is important. With HIV self-testing being a novel strategy as well, despite little research on the uptake and outcomes of HIV self-testing among women, we analyzed and presented data from GetaKit.ca on cis gendered women. Our findings showed less HIV access among women, despite ongoing and historical risk and equal positivity rates on testing compared to cis gendered men. We take these findings to mean that we need more access to HIV testing for women and we see online platforms like GetaKit.ca as one potential strategy to achieve this outcome. Indeed, from our work, GetaKit.ca increased testing rates and did reach women with undiagnosed HIV infections. While many questions remain unanswered about such digital health solutions, it appears that they should be added as core health services. If we wish to achieve the UNAIDS 95-95-95 targets by 2030, investment in such platforms might be required.

## Supplemental Material

sj-docx-1-whe-10.1177_17455057251322810 – Supplemental material for HIV self-testing in cis women in Canada: The GetaKit study Supplemental material, sj-docx-1-whe-10.1177_17455057251322810 for HIV self-testing in cis women in Canada: The GetaKit study by Lauren Orser, Alexandra Musten, Hannah Newman, Molly Bannerman, Marlene Haines, Jennifer Lindsay and Patrick O’Byrne in Women’s Health

sj-docx-2-whe-10.1177_17455057251322810 – Supplemental material for HIV self-testing in cis women in Canada: The GetaKit study Supplemental material, sj-docx-2-whe-10.1177_17455057251322810 for HIV self-testing in cis women in Canada: The GetaKit study by Lauren Orser, Alexandra Musten, Hannah Newman, Molly Bannerman, Marlene Haines, Jennifer Lindsay and Patrick O’Byrne in Women’s Health

## Declarations

**Ethics approval and consent to participate:** Ethics approval for this study was obtained from the University of Ottawa Research Ethics Board (H-12-20-6450). All GetaKit.ca participants had to visit the study website, review and digitally sign the online consent forms, register using a name and contact information, complete an online HIV risk assessment, consent to obtaining the self-test, and place their order. Parent or guardian consent was not applicable as this study did not involve minors.

**Consent for publication:** As part of completing the online research consent form, all participants provided consent for anonymized data to be included in research publications.

**Availability of data and materials:** Data available upon request.
