# Supplementary material for: HIV self-testing in cis women in Canada: The GetaKit study
Source: Womens Health (Lond). 2025 Mar 31;21:17455057251322810. doi: 10.1177/17455057251322810 (PMC11960179; doi:10.1177/17455057251322810)
Supplement: sj-docx-1-whe-10.1177_17455057251322810 – Supplemental material for HIV self-testing in cis women in Canada: The GetaKit study [file sj-docx-1-whe-10.1177_17455057251322810.docx]

Supplementary Appendix 1

GetaKit Questionnaire

1. Do you have symptoms now or in the past two weeks?
2. In the last 4 weeks, did you have sex with anyone who was newly diagnosed with HIV or an STI?
3. Are you currently taking HIV pre-exposure prophylaxis (PrEP)?
4. What is/are the sex(es) of the people you have sex with?
5. What are the risk factors of your sexual partners?
6. What is your current genitalia?
7. What are your sex practices?
8. Are you pregnant or risk for pregnancy?^*^
9. Do you engage in sex work?
10. Have you ever been tested for HIV or STIs?

If yes:

- How long ago was your last HIV test?
- How long ago was your last STI test?
- Have you ever been diagnosed with any STIs, including HIV?
- If prior diagnosis of chlamydia or gonorrhea, how long ago was your treatment?

1. Why are you re-testing for STIs/HIV today?
2. Have you ever used or shared drug equipment? (This includes needles, cookers, filters, bills/straws to snort drugs, or pipes/stems to smoke crack or meth).
3. Do you have a health card or other form of coverage/insurance that you could use for testing?
4. Would you be willing to go to a laboratory with your health insurance card for testing? (This does not apply to the HIV self-test).

^* Only applies to persons who report current genitalia as vagina, fronthole, internal genitals^
